# Pharmacotherapy Literacy and Parental Practice in Use of Over-the-Counter Pediatric Medicines

**DOI:** 10.3390/medicina55030080

**Published:** 2019-03-26

**Authors:** Dušanka Krajnović, Stana Ubavić, Nataša Bogavac-Stanojević

**Affiliations:** 1 Department of Social Pharmacy and Pharmaceutical Legislation, Faculty of Pharmacy, University of Belgrade, 11221 Belgrade, Serbia; 2 Medicines and Medical Devices Agency of Serbia (ALIMS), 11221 Belgrade, Serbia; stana.ubavic@alims.gov.rs; 3 Department of Medical Biochemistry, Faculty of Pharmacy, University of Belgrade, 11221 Belgrade, Serbia; naca@pharmacy.bg.ac.rs

**Keywords:** parents, over-the-counter, antipyretics, pharmacotherapy, pre-school children, pharmacist, pharmacotherapy literacy

## Abstract

*Background and objectives:* Pharmaceutical literacy skills of parents are crucial for appropriate and safe medication use in pre-school children (ages 1–7 years). A recent study on pharmacotherapy literacy from Serbia showed that one in five parents have difficulty understanding common information about the use of medicines. Because antipyretics are considered to be the most frequently used group of over-the-counter (OTC) medications during the pre-school period, we aimed to: (i) examine parental practice and expectations in antipyretic medication use, and (ii) analyze associations of parental practice and expectations related to socio-economic status and pharmacotherapy literacy. *Materials and methods:* A cross-sectional survey using a self- report validated specific instrument was conducted with the parents of pre-school children in kindergartens in Belgrade, Serbia. Pharmacotherapy literacy refers to the knowledge and personal skills needed to meet the complex demands of medicine use in both healthcare and non-healthcare settings. A comprehensive literature review, expert-focus group consultation, and pre-testing were employed in 4-item multiple-choice test development to explore practice and expectations related to the use of OTC pediatric antipyretic medicines. *Results:* The final analytical cohort was comprised of 813 participants, the majority (63.3%) chose a medicine based on a physician’s suggestion and only 15.4% of parents reported they would follow the advice of a pharmacist. More than a half of parents (54.1%) would need advice about antipyretic medicine from a pharmacist, firstly in a simpler language. Parents satisfied with the information given by a pharmacist had higher pharmacotherapy literacy, compared to parents with lower levels (OR–0.718, 95%CI (0.597–0.865), *p* < 0.001). Men had a higher expectation of pharmacists to explain medicine use in a simpler language (OR–1.630, 95%CI (1.063–2.501), *p* = 0.025), as well as parents with three or more children (OR–2.527, 95%CI (1.43–4.459), *p* = 0.001). Parents with higher knowledge about medicine use were less likely to ask for simpler information (OR–0,707; 95%CI (0,583–0,856), *p* < 0,001). *Conclusions:* Our main finding is that practice in antipyretic OTC medicine use was associated with levels of parental pharmacotherapy literacy. The expectations of pharmacists were higher among parents with lower levels of pharmacotherapy literacy, who expected more information in a simpler and more precise language. This study highlighted the need for pharmacists to identify risks in parental practice and to provide information about medicines to parents of pre-school children in a simpler and more appropriate way.

## 1. Introduction

Parental pharmacotherapy literacy plays a very important role in the management of childhood illnesses, as it contributes to their future decisions, expectations, and practice with medicine use [1]. Koster et al. highlight that pharmacotherapy literacy is a complex concept requiring different skills in order to ensure good and safe use of medication [2], and to emphasize the need to identify patients with limited health literacy skills in order to prevent problems with the use of medicines [1]. Pharmacotherapy literacy is context and content specific [3]. In the case of medication use, pharmacotherapy literacy skills that include numeracy, literacy, and knowledge are crucial for appropriate and safe medication.

Parental practice toward the use and choice of antipyretic medicines for their children also plays an important role in adherence [4]. Beliefs can also have an influence on the behavior, practice, and expectations of children in the future, as they may act with similar patterns to their parents.

Former studies on parental practice and parental expectations have been focused on special types of medicines given for illnesses such as asthma or ADHD (Attention Deficit Hypersensitivity Disorder) [5,6]. These results support the hypothesis that parental beliefs and adherence to prescribed therapy are related. Furthermore, practice in medicines’ use was mostly examined among adults [6,7].

Recent studies suggest that the most frequently used over-the-counter medicines (OTCs) in the pediatric population are antipyretics (about 60%) [4,8]. According to a study conducted in Australia, 80% of adults and 40% of children use OTC medicines every month [9]. Research in the USA has shown that about 80% of those who took OTCs have done so for pain relief, and half consumed them for relief of cold symptoms (fever, cough, sore throat) [9].

Several studies have also suggested that parents have poor knowledge about antipyretics. For instance, every third parent thinks that antipyretic should be given for every fever case [9]. Our study on pharmacotherapy literacy among parents of pre-school children in Serbia has shown that every fifth parent has difficulty understanding information that is given either in written or spoken form about the use of medicines [10]. Unintentional errors in the use of pediatric medicines made by parents can be very frequent and could be due to lack of knowledge and low levels of pharmacotherapy literacy [6,7]. Most errors by parents are made due to a failure to adequately calculate and understand dosage regimens or wrong measurements with included devices [11,12,13]. These errors are often related to liquid pediatric medicines, mainly antipyretics [14,15]. Socio-demographic characteristics, such as number of children, parental age, and level of education were reported to be factors that influenced the usage of antipyretic medicines [10].

Currently, there is a specific gap in parental pharmacotherapy literacy levels and associations of their practice in antipyretic OTC use for children.

As parents are responsible for their children’s medication outcomes, and often make errors during the administration of antipyretic medicines [11,12,13], we aimed to examine parental practice towards the use of antipyretic pediatric medicines and their expectations from pharmacists in that respect. Firstly, we investigated parental sources of information and choices of medicines and examined how these sources were related to the results of pharmacotherapy literacy. 

## 2. Materials and Methods

### 2.1. Design and Settings

The design of the survey was cross-sectional, using a self- report validated specific instrument, and the research was conducted as part of a larger study on pharmacotherapy literacy among parents and caregivers of pre-school children in Serbia [10].

### 2.2. Data Collection

Data were collected from May to October 2016 at 10 kindergartens in a number of municipalities in the Serbian city of Belgrade using the same sample of parents from our previous study on pharmacotherapy literacy among parents [10]. The appropriate informed consent to participate in the study was given by participants. Ethics approval for this research was received from the Committee for Biomedical Research Faculty of Pharmacy, Belgrade (321/2, approved on 15 March 2016).

The questionnaire was distributed to parents at the regularly scheduled parent-teacher meetings in the kindergarten and administered by trained interviewers.

The questionnaire was about the practice and expectations in the use of OTC pediatric medicines and was submitted together with the PTHL-SR questionnaire (Pharmacotherapy literacy questionnaire in Serbian) [10] and personal background questions. The questionnaire about practice and expectations in OTC pediatric medicines was constructed by experts in a focus group interview during the construction of the pharmacotherapy literacy questionnaire in Serbian. The detailed development and psychometric testing of the PTHL-SR questionnaire was published elsewhere [16]. After two cycles of focus group engagement [17,18], 4 questions were made as a tool for the quick screening of the common practices and expectations of parents towards OTC medicines’ use. We used antipyretic medicines as an example of OTC pediatric medicines that most of the parents were likely to be familiar with [11,13], and because antipyretics are considered to be the most frequently used group of medicines [4,8].

The questionnaire about practice with, and expectations of, OTC pediatric medicines had 4 questions:

1. What is your preference for choice of antipyretic pediatric medicine?
2. What could pharmacists do to improve a patient’s knowledge about antipyretic medicine use?
3. Where do you find information about medicine use and their effects?
4. Who gives you information about what kind of medicine to give a child?

Answers to questions were in a multiple choice format, and for every question the respondent could choose just one answer.

The PTHL-SR questionnaire filled out by participants was administered in Serbian and included 14 items within 4 domains of pharmacotherapy literacy: knowledge, understanding, numeracy, and access to medicine-related information [10]. We have calculated the percentage of correct answers for each, as well as the percentage of correct answers within domains of pharmacotherapy literacy.

The total result in PTHL-SR for each participant is the percentage of correct answers. Moreover, we divided achieved results into three groups: the first group included results with up to 8 (64%) correct answers (low level in PTHL-SR), the second group included results between 9 and 10 (65–85%) of correct answers (medium PTHL-SR level), and the third group included results between 11 and 14 (86–100%) of correct answers (high PTHL-SR level) [10].

Information on personal background with demographic, socio-economic characteristics, and behavioral patterns were obtained through self-reported questionnaires.

### 2.3. Statistical Analysis

The statistical testing of group differences for categorical variables was examined by the chi-square test of independence. Calculated scores for knowledge, understanding of information, numeracy skills, and total PTHL-SR were compared between the groups by the student’s *t*-test for two samples and a one-way analysis of variance (ANOVA) with a post hoc Tuckey-Kremer test. Using binary univariate logistic regression analysis, we determined the probability of socio-demographic characteristics to predict answers to questions about expectations. In addition, we performed multivariate logistic regression analysis to determine independent predictors for the same answers. We included only statistically significant predictors from univariate logistic regression in analysis. A two-tailed *p* value of *p* ≤ 0.05 was considered significant. All calculations were performed using SPSS, version 22.0 (IBM Corp., Armonk, NY, USA).

Demographic and socio-economic characteristics included in this analysis were: age (in years), gender (women, men), education (elementary or secondary school and university degree), marital status (living with an intimate partner/living alone), chronic illnesses of children, employment, and smoking status (smoker/non-smoker). Behavior patterns that were analyzed included: breastfeeding and number of annual visits to a physician. The self-estimation of a parent’s health status was assessed as average, bad, good, or excellent.

## 3. Results

This study was done on a sample of 813 parents, mainly females (81.30%), between 30 to 40 years of age (70.85%) with two children (56.70%).

Most of parents (63.3%) said that they choose antipyretic medicine for fever symptoms based on a suggestion by a physician. Only 15.4% of parents would appreciate the advice from a pharmacist. Positive previous experiences would make every fifth parent choose the same antipyretic medicine again.

When asked what they think pharmacists in the pharmacy should do in order to improve parental knowledge about the use of medicine for children, 44.9% of parents answered that they receive all necessary information from a pharmacist. However, half of parents think that pharmacist should explain information: with simpler words (18.5%) or write information on about medicines’ use on paper (10.4%), while every fourth parent (26.2%) had the opinion that it is necessary to communicate with patients and provide information in both written and spoken formats. The majority of our respondents (80.8%) stated that they find all the necessary information about use and efficacy in the Patient Information Leaflet (PIL), 4.1% of parents would first search for information on the outer packaging, and 15.1% of parents answered that they do not read the leaflet but instead ask for information from a pharmacist or physician. When asked whom they would ask about which OTC antipyretic medicine to give to a child, almost all of the parents in our study (94.9%) said that they would ask a pharmacist or physician.

Socio-demographic differences and the results in Table 1 show that there are no significant socio-demographic parameters related to the parental choice of OTC antipyretic medicines.

Independent of the self-estimated health levels, nearly the same number of parents (more than 60%) choose OTC antipyretic medication based on a physician’s advice (*p* = 0.746). Moreover, it was shown that parents, who received information about medicines from different sources (58.5%), were more likely to choose medicine based on previous positive experiences than those who received information from a physician or pharmacist (18.9%), *p* < 0.001 (Table 1).

The socio-demographic characteristics of respondents, and their answers to the question about what they would expect a pharmacist to do in the pharmacy in order to improve parental knowledge about the use of OTC medicines for children, are shown in Table 2.

Every fourth male parent (26.6%) expected information about OTC medicines to be given in a simpler language by a pharmacist, while only 16.6% women expected the same (*p* = 0.006). Also, almost half of women (47.4%) stated that pharmacists give them all the necessary information about OTC medicines for their children. The analysis revealed that there was no difference between groups, i.e., access to information about medicine use is not related to practice or expectations from pharmacists (*p* = 0.421), Table 2.

The youngest parents (18–29 years) expected information about medicine use to be given in a simpler language (25.4%), in comparison to older parents group (30–40 years: 17.9%, 41–50 years: 18.0%).

We also estimated self-assessment of health and its relation to differences in parental practice and expectations for pharmacists to provide information about the use, for children, of OTC medicines. Independent of self-estimated health levels, almost half of the parents (40–50%) reported that pharmacists always provided sufficient information.

There is no significant difference in socio-demographic parameters based on access to information about the use of medicines (Table 3).

In the part of the self-assessment of health and its relation to medicine use, information, and access, nearly the same majority of parents (more than 80%) find information about medicines use in the Patient Leaflet Instruction (*p* = 0.545).

While, most parents find information about medicine use in the Patient Leaflet Instruction (78.3%—males; 81.4%—females), 15.1% of parents expect information about medicine use from the pharmacist, i.e., they do not read Patient Leaflet Instructions.

Subsequently, we compared parental practice and expectations about OTC use according to pharmacotherapy literacy levels estimated with PTHL-SR. The results are shown in Table 4.

Parents who stated that pharmacists should explain and write information about medicine use in a simpler way in order to improve parental knowledge and those who stated that they always get all necessary information from a pharmacist had significantly higher knowledge and a higher total PTHL-SR score than parents who declared that pharmacists should only explain information about medicine use in a simpler language. Parents who find information about the use and efficacy of the medicine in the Patient Leaflet Instruction had a higher total score PTHL-SR than parents who find information on the outer packaging or internet). In addition, parents who received recommendations about which kind of OTC antipyretic medicine to give to a child from a physician or a pharmacist had a higher knowledge score but a lower numeric and total PTHL-SR score than parents who receive information from other sources of information (e.g., family members, newspapers, internet).

We evaluated socio-demographic predictors for parents to ask for simpler information (Table 5). Compared to females, male parents have a two times higher probability to ask for information to be given in a simpler way from a pharmacist. Our study has also shown that parents with three or more children, ask for simpler information from pharmacists much more often than parents with only one child (OR–2.262). Parents who visited a physician more times annually had a lower probability to ask for simpler information: (1–2 times: OR–0.604; 3–4 times: OR–0.582,) than parents who did not visit a physician.

Additionally, we examined if total PTHL-SR levels and results according to domains of pharmacotherapy literacy influenced the probability that a parent will need simpler information from the pharmacist. It is shown that lower results in knowledge and lower total PTHL-SR levels increased the probability that a parent would expect simpler information from the pharmacist.

Finally, parameters significant in univariate logistic regression analysis were included in the multiple logistic regression analysis with the aim to detect independent predictors for asking simpler information about medicines. The male sex is connected to a higher probability for expectations that a pharmacist should explain medicine use in a simpler language (OR–1.630, 95%CI (1.063–2.501), *p* = 0.025). This probability is similar for parents with three and more children (OR–2.527, 95%CI (1.432–4.459), *p* = 0.001) who also need simpler and clearer information, in comparison to parents with one child. On the other side, the number of annual visits to a pediatrician (1–2 times: OR–0.587, 95%CI (0.380–0.910), *p* = 0.017; 3–4 times: OR–0.527, 95%CI (0.314–0.885), *p* = 0.015) relate to a lower probability that parents will ask for precise information from a pharmacist. If parents have higher knowledge about medicine use, there is a lower probability that they will ask for simpler information (OR–0.707; 95%CI (0.583–0.856), *p* < 0.001). 

## 4. Discussion

In our study, we examined parental practices and expectations towards antipyretic medicine use and, for the first time, its relationship to parental pharmacotherapy literacy [10]. Furthermore, we examined parental expectations related to information about the use of medicines given by healthcare professionals, especially pharmacists. In Serbia, research about attitudes and practices related to use of herbal medicines was carried out with adults [19]. There is, however, only a very limited body of work about the parental practice of OTCs and pharmacotherapy literacy to which we can compare our findings. Regarding parental practice about medicine use, a study about parental knowledge, attitudes, and behaviors towards children with epilepsy was made in Belgrade (Serbia) by Gazibara et al. (2014) [20]. This study highlighted that it is necessary to ensure education and support for both parents and children.

However, more than half of parents (54.1%) agreed that they need advice about antipyretic medicine use and recommendations from the pharmacist in a simpler and more precise language. This is in line with the traditional role of the pharmacist as an advisor to patients and indicates that pharmacists should increase their communication competence and learn to identify parents who need more clarification and explanation. As suggested by respondents in our study, information should be presented in a simpler way, with language that is easy to understand for parents with low levels of pharmacotherapy and health literacy.

Although most parents knew that the information about medicines was presented in the Patient leaflet Instruction, more males (26.6%) than females (16.6%) reported that they would need simpler information from the pharmacist, rather than just reading the Patient Leaflet Instructions. That finding is in line with results of pharmacotherapy literacy among parents in Serbia, which showed lower results in pharmacotherapy literacy among males than females. Moreover, this result suggests that parents who read instructions understood written information and had higher levels of pharmacotherapy literacy.

Our study found that parental age was one of the key factors that contributed to the need for information about medicine use to be given by pharmacist in a simpler language. This need is mostly present among younger parents (18–29 years, 25.4%, *p* < 0.05), mainly because of lack of experience. However, an unusual finding in our study is that parents with three and more children, although having the best results for empirical knowledge in PTHL-SR, were two times likelier to ask for information from the pharmacist in a simpler language. A possible explanation could be that those parents may have less free time for reading Patient leaflet instructions. According to our previous investigation, older parents with more children had the best parental pharmacotherapy literacy results [10]. Birchley and Conroy [21] identified that a parent’s age, number of children, and employment status are related to the frequency of use and choice of antipyretic OTC medicine for children. Our study has also showed that parental PTHL-SR levels are related to their expectations concerning the use of OTC medicines for children. Parents with higher PTHL-SR level were satisfied with the information that the pharmacist provided, while parents with lower levels stated that information should be simpler and that they needed more information and recommendations. Parents with higher levels of pharmacotherapy literacy are likely to have greater critical thinking abilities, which makes them able to make decisions on their own. They also know how to use their knowledge and are satisfied if they read Patient Leaflet Instructions. This is an important finding because the level of parental pharmacotherapy literacy has been associated with access to information about medicine use, i.e., parents with lower levels of pharmacotherapy literacy rely more on the pharmacist, while parents with adequate levels of pharmacotherapy literacy are able to read information from the Patient Leaflet Instructions in order to self-administer medicines in a proper way.

Findings from our study are significant with regard to patients who need more assistance in the administration of OTC medicines for pre-school children, especially among parents with different levels of pharmacotherapy literacy. Younger parents, men, and families with three and more children are more likely to need simpler information and more communication guidance from the pharmacist. These are also risk groups for misunderstanding information and for increased errors in OTC pediatric medicine use. A recent study from the Netherlands also suggested that the most useful way to support patients who need more explanation is additional written information (e.g., pictograms, animations, or verbal information), information suitable to the patient’s needs, information that is easily visible (e.g., use of larger font size, shorter sentences), and information that is easily accessed (e.g., an illustrated card) [22]. Koster et al. [23] have developed a RALPH interview guide (Recognizing and Addressing Limited Pharmaceutical Literacy) that provides guidance on the level of skill needed for good medication use and is more suitable for use in a medication specific context, such as a community pharmacy. With this insight, pharmacists could recognize parents with limited pharmacotherapy literacy, and, as proposed in the described conceptual model of health literacy, pharmacotherapy literacy should act as a risk and is important in order to reduce barriers in communication [24].

### Limitations

There are potential limitations to our study. First, we made screening questions only about practices and sources of information about OTC antipyretic medicine use. Further investigation should include concise questions in relevant health circumstances linked with clinical outcomes. Second, it is unclear if parental practice in the use of OTC Pediatric Medicines would differ if parents were to come from non-urban areas and villages. Thus, it is possible that current findings may not be generalizable. However, the large sample size and inclusion of parents from different socio-demographic groups are major strengths of this study and increase the generalization of the findings. It is not investigated if the educational background of the parents could have an impact, or if our findings were due, in part at least, to a sampling bias, whereby those parents who were health care professionals by background could have different, and perhaps better, approaches to OTC medicines than other parents with different jobs or knowledge. Another limitation is the use of a cross-sectional study design, which makes it difficult to identify causal relationships. We suggest a longitudinal study design in the future to determine causality.

## 5. Conclusions

As self-medication is becoming increasingly common and patients with limited pharmacotherapy literacy are at increased risk of medication-related problems, we have provided insight into parental pharmacotherapy literacy in Serbia and its association with parental practices and expectations about OTC use.

The findings indicate that parental practice and expectations about OTC use are associated with a parent’s level of PTHL. The questions from the instrument used in our study are phrased in such a way that they can also be used in healthcare settings, so in the future, they could be used as a tool to assist pharmacists in identifying patients’ literacy concerns at the point of service. The present study suggests the need for pharmacists to identify risks in parental practice and to provide information about medicines to parents of pre-school children in a more appropriate way. Pharmacotherapy literacy enhancing interventions, especially those that could be performed by pharmacists, who are the most available healthcare providers at community level, are needed for good and safe medication use. Further research could expand the efforts to provide tailored pharmaceutical care for parents of pre-school children in the future.

## Figures and Tables

**Table 1:** The choice of over-the-counter (OTC) antipyretics medicines according to parental socio-demographic characteristics, behavioral, patterns and parental access to information.

Socio-Demographic Characteristics		Previous Positive Experience % (*N*)	Advice from Physician % (*N*)	Advice from Pharmacist % (*N*)	Statistics
Gender	Male	21.5 (32)	65.1 (97)	13.4 (20)	chi-square = 0.576; df = 2; *p* = 0.750
	Female	20.8 (137)	63.3 (418)	15.9 (105)	
Age (years)	18–29	18.6 (11)	66.1 (39)	15.3 (9)	chi-square = 5.60; df = 4; *p* = 0.229
	30–40	22.4 (128)	63.6 (364)	14.0 (80)	
	41–50	16.9 (30)	62.9 (112)	20.2 (36)	
Number of children	One child	20.7 (50)	63.2 (153)	16.1 (39)	chi-square = 0.142; df = 4; *p* = 0.998
	Two children	21.1 (97)	63.7 (293)	15.2 (70)	
	Three children and more	20.6 (22)	64.5 (69)	15.0 (16)	
Marital status	Single parent ^c^	27.5 (22)	57.5 (46)	15.0 (12)	chi-square = 2.40; df = 2; *p* = 0.302
	Married ^d^	20.2 (147)	64.3 (469)	15.5 (113)	
Education	University degree and higher ^a^	21.1 (96)	64.0 (292)	14.9 (68)	chi-square = 0.233; df = 2; *p* = 0.890
	Middle school and less education ^b^	20.7 (73)	63.2 (223)	16.1 (57)	
Employment	Employed	21.8 (155)	63.2 (450)	15.0 (107)	chi-square = 3.08; df = 2; *p* = 0.214
	Unemployed	14.4 (14)	67.0 (65)	18.6 (18)	
Chronic disease of a child	No	20.1 (142)	64.9 (458)	15.0 (106)	chi-square = 3.57; df = 2; *p* = 0.168
	Yes	26.2 (27)	55.3 (57)	18.4 (19)	
Smoking	No	29.8 (112)	64.6 (366)	15.7 (89)	chi-square = 1.55; df = 2; *p* = 0.460
	Yes	23.7 (57)	61.4 (148)	14.9 (36)	
Breast feeding of a first child	No	19.0 (16)	65.5 (55)	15.5 (13)	chi-square = 0.21; df = 2; *p* = 0.901
	Yes	21.1 (153)	63.4 (459)	15.5 (113)	
Annual visits to pediatrician	1–2 times	21.3 (57)	63.4 (170)	15.3 (41)	chi-square = 1.06; df = 4; *p* = 0.930
	3–4 times	22.5 (59)	62.2 (163)	15.3 (40)	
	5 times and more	19.0 (53)	65.2 (182)	15.8 (44)	
Access to information about medicines *	From physician or pharmacist	18.9 (145)	65.8 (505)	15.4 (118)	chi-square = 39.0; df = 2; *p* < 0.001
	Other sources of information	58.5 (24)	24.4 (10)	17.1 (7)	

^a^ University degee and higher (at least 16 years of education). ^b^ Middle school or less (8–12 years of education). ^c^ Single parent (living alone with a child—divorced, widow). ^d^ Married (living with a partner). * Question related to access to information about medicines by parents. Df—Degrees of freedom.

**Table 2:** Differences in parental opinions about role of pharmacists according to parental socio-demographic, characteristics, behavioral patterns, and parental access to information.

Socio-Demographic Characteristics		Pharmacists Should Explain Information in a Simpler Language % (*N*)	Pharmacists Should Write Information in a Simpler Way % (*N*)	Pharmacists Should Explain and Write Information in a Simpler Way % (*N*)	Pharmacist Always Give All Necessary Information % (*N*)	Statistics
Gender	Male	26.3 (40)	13.2 (20)	26.3 (40)	34.2 (52)	chi-square = 12.33; df = 3; *p* = 0.006
	Female	16.6 (110)	9.8 (65)	26.2 (173)	47.4 (313)	
Age (years)	18–29	25.4 (15)	11.9 (7)	18.6 (11)	44.1 (26)	chi-square = 12.97; df = 6; *p* = 0.044
	30–40	17.9 (103)	10.8 (62)	24.1 (139)	47.2 (272)	
	41–50	18.0 (32)	9.0 (16)	35.4 (63)	37.6 (67)	
Number of children	One child	14.7 (36)	11.4 (28)	30.2 (74)	43.7 (107)	chi-square = 11.24; df = 6; *p* = 0.081
	Two children	18.2 (84)	10.6 (49)	25.2 (70)	46.0 (212)	
	Three children and more	28.0 (30)	7.5 (8)	21.5 (23)	43.0 (46)	
Marital status	Single parent ^c^	18.8 (15)	13.8 (11)	25.0 (20)	42.5 (34)	chi-square = 1.09; df = 3; *p* = 0.780
	Married ^d^	18.4 (135)	10.1 (74)	26.3 (193)	45.2 (331)	
Education	University degree and higher ^a^	18.8 (15)	13.8 (11)	25.0 (20)	42.5 (34)	chi-square = 0.66; df = 3; *p* = 0.883
	Middle school and less education ^b^	18.4 (135)	10.1 (74)	26.3 (57)	45.2 (331)	
Employment	Employed	19.0 (136)	10.3 (74)	27.0 (193)	43.7 (313)	chi-square = 4.22; df = 3; *p* = 0.239
	Unemployed	14.4 (14)	11.3 (11)	20.6% (20)	53.6 (52)	
Chronic disease of a child	No	12.6 (13)	5.8 (6)	33.0 (34)	48.5 (50)	chi-square = 7.03; df = 3; *p* = 0.071
	Yes	19.3 (137)	11.1 (79)	25.2 (179)	44.4 (315)	
Smoking	No	18.6 (106)	11.8 (67)	26.0 (148)	43.7 (249)	chi-square = 3.65; df = 3; *p* = 0.302
	Yes	18.2 (44)	7.4 (18)	26.9 (65)	47.5 (115)	
Breast feeding of a first child	No	23.5 (20)	15.3 (13)	16.5 (14)	44.7 (38)	chi-square = 6.88; df = 3; *p* = 0.075
	Yes	17.9 (130)	9.9 (72)	27.3 (199)	44.9 (327)	
Annual visits to pediatrician	1–2 times	24.4 (66)	9.6 (26)	23.3 (63)	42.6 (115)	chi-square = 10.2; df = 6; *p* = 0.411
	3–4 times	16.3 (43)	11.0 (29)	27.0 (71)	45.6 (120)	
	5 times and more	14.6 (41)	10.7 (30)	28.2 (79)	46.4 (130)	
Access to information about medicines*	From physician or pharmacist	18.2 (140)	10.1 (78)	26.5 (204)	45.2 (348)	chi-square = 2.81; df = 3; *p* = 0.421
	Other sources of information	23.3 (10)	16.3 (7)	20.9 (9)	39.5 (17)	

^a^ University degee and higher (at least 16 years of education). ^b^ Middle school or less (8–12 years of education). ^c^ Single parent (living alone with a child—divorced, widow). ^d^ Married (living with a partner). * Question related to access to information about medicines by parents. Df—Degrees of freedom.

**Table 3:** Parental socio-demographic differences related to medicine use information access.

Socio-Demographic Characteristics		On the Outer Packaging/Internet % (*N*)	In the Patient Leaflet Instruction % (*N*)	Parents Would Expect that Pharmacists Give Information at Pharmacy% (*N*)	Statistics
Gender	Male	6.6 (10)	78.3 (119)	15.1 (23)	chi-square = 3.07; df = 2; *p* = 0.215
	Female	3.5 (23)	81.4 (538)	15.1 (100)	
Age (years)	18–29	3.4 (2)	4.2 (24)	3.9 (7)	chi-square = 1.69; df = 4; *p* = 0.793
	30–40	86.4 (51)	80.7 (465)	79.2 (141)	
	41–50	10.2 (6)	15.1 (87)	16.9 (30)	
Number of children	One child	5.3 (13)	2.8 (13)	6.5 (7)	chi-square = 6.44; df = 4; *p* = 0.169
	Two children	78.8 (193)	82.3 (375)	83.2 (89)	
	Three children and more	15.9 (39)	15.8 (73)	10.3 (11)	
Marital status	Single parent	5 (4)	81.3 (65)	13.8 (11)	chi-square = 0.31; df = 2; *p* = 0.858
	Married ^d^	4 (29)	80.8 (592)	15.3 (112)	
Education	University degree and higher ^а^	4.6 (21)	78.3 (360)	17.2 (79)	chi-square = 0.45; df = 2; *p* = 0.108
	Middle school and less education ^b^	3.4 (12)	84.1 (297)	12.5 (44)	
Employment	Employed	4.2 (30)	80.9 (579)	14.9 (107)	chi-square = 0.39; df = 2; *p* = 0.822
	Unemployed	3.1 (3)	80.4 (78)	16.5 (16)	
Chronic disease of a child	No	4.2 (30)	81.0 (575)	14.80 (105)	chi-square = 0.83; df = 2; *p* = 0.660
	Yes	2.9 (3)	79.6 (82)	17.5 (18)	
Smoking	No	4.0 (23)	80.0 (456)	16.0 (91)	chi-square = 0.99; df = 2; *p* = 0.608
	Yes	4.1 (10)	82.6 (200)	13.2 (32)	
Breast feeding of a first child	No	2.4 (2)	4.8 (29)	1.7 (2)	chi-square = 3.14; df = 2; *p* = 0.208
	Yes	21.1 (83)	63.4 (580)	15.5 (117)	
Annual visits to pediatrician	1–2 times	3.3 (9)	80.0 (216)	16.7 (45)	chi-square = 6.40; df = 4; *p* = 0.170
	3–4 times	3.8 (10)	85.2 (224)	11.0 (29)	
	5 times and more	5.0 (14)	77.5 (217)	17.5 (49)	
Access to information about medicines *	From physician or pharmacist	3.8 (29)	81.3 (626)	14.9 (115)	chi-square = 3.86; df = 2; *p* = 0.145
	Other sources of information	9.3 (4)	72.1 (31)	18.6 (8)	

^a^ University degee and higher (at least 16 years of education). ^b^ Middle school or less (8–12 years of education). ^c^ Single parent (living alone with a child—divorced, widow). ^d^ Married (living with a partner). * Question related to access to information about medicines by parents. Df—Degrees of freedom.

**Table 4:** Parental practice and expectations about OTC pediatric medicines use and Pharmacotherapy literacy. PTHL-SR, Pharmacotherapy literacy questionnaire in Serbian.

Answers to Questions about Practice and Expectations	Knowledge X ± SD (Max Score: 5)	Statistics	Understanding X ± SD (max score: 3)	Statistics	Numeracy X ± SD (Max Score: 5)	Statistics	Total Score PTHL-SR X ± SD (Max Score: 14)	Statistics
Question 1 (What is your preference for choice of antipyretic pediatric medicine?)								
Previous positive experience	3.81 ± 0.96	F = 0.925; df _(2.806)_ *p* = 0.395	2.44 ± 0.85	F = 0.397; df _(2.806)_ *p* = 0.672	3.86 ± 0.90	F = 0.053; df _(2.806)_ *p* = 0.948	11.85 ± 2.02	F = 0.708; df _(2.806)_ *p* = 0.493
Advice from physician	3.90 ± 0.87		2.50 ± 0.87		3.83 ± 0.92		12.04 ± 1.84	
Advice from pharmacist in pharmacy	3.81 ± 0.98		2.46 ± 0.83		3.82 ± 0.98		11.91 ± 2.25	
Question 2 (What could pharmacist do to improve patient’s knowledge about antipyretic medicines use?)								
To explain information about medicines use in a simpler language	3.63 ± 0.94	F = 6.129; df _(3.809)_ *p* < 0.001	2.39 ± 0.95	F = 1.816; df _(3.809)_ *p* = 0.143	3.73 ± 0.95	F = 1.328; df _(3.809)_ *p* = 0.264	11.53 ± 2.05	F = 3.842; df _(3.809)_ *p* < 0.001
To write information about medicines use in a simpler way	3.71 ± 0.88		2.64 ± 0.91		3.79 ± 0.85		11.89 ± 1.90	
Both. To explain it simple and to write information	3.93 ± 0.96 *		2.53 ± 0.83		3.91 ± 0.98		12.16 ± 2.04*	
I always get all necessary information from pharmacist	3.96 ± 0.87 *		2.47 ± 0.84		3.86 ± 0.93		12.09 ± 1.82*	
Question 3 (Where do you find information about medicines use and their effects?)								
On the outer packaging/Internet	3.58 ± 1.12	F = 2.008; df _(2.810)_ *p* = 0.135	2.24 ± 0.90	F = 1.742; df _(2.810)_ *p* = 0.176	3.64 ± 1.13	F = 1.037; df _(2.810)_ *p* = 0.355	11.12 ± 2.57	F = 4.039; df _(2.810)_ *p* = 0.018
In Patient Leaflet Instruction	3.87 ± 0.90		2.51 ± 0.86		3.86 ± 0.91		12.05 ± 1.87*	
I do not read it, I expect an advice in pharmacy	3.94 ± 0.93		2.44 ± 0.89		3.80 ± 0.97		11.85 ± 2.09	
Question 4 (Who gives you an information which kind of OTC medicine to give a child?)								
Physician/Pharmacist	4.05 ± 0.87	F = 5.990; df _(1.811)_ *p* = 0.015	2.35 ± 0.83	F = 3.569; df _(1.811)_ *p* = 0.059	3.60 ± 0.98	F = 10.930; df _(1.811)_ *p* = 0.001	10.93 ± 1.74	F = 47.91; df _(1.811)_ *p* < 0.001
Other sources of information (family members, newspapers, Internet)	3.83 ± 0.93		2.51 ± 0.87		3.89 ± 0.91		12.18 ± 1.91	

* vs first group (*p* < 0.05); Df—Degrees of freedom.

**Table 5:** Socio-demographic characteristics of parents and PTHL-SR level as predictors for asking for simpler information about medicine use.

Socio-Demographic Characteristics	Wald	OR	95% CI	*p*-Value
Gender				
Men	7.546	1.789	1.181–2.709	0.006
Age				
18–50 years	0.743	0.860	0.610–1.212	0.389
Number of children				
2 children	1.406	1.294	0.845–1.979	0.236
3 children and more	8.445	2.262	1.304–3.922	0.004
Marital status				
Single parent	0.005	1.022	0.566–1.847	0.942
Education				
Middle school or less	0.116	0.940	0.658–1.343	0.733
Employment				
Employed	1.172	1.390	0.766–2.524	0.279
Smoking				
Smokers	0.019	0.973	0.659–1.435	0.106
Breast feeding of a first child				
Yes	1.289	0.813	0.569–1.435	0.889
Self-estimation of health status				
Bad	0.066	1.038	0.779–1.383	0.797
Chronic illness of a child				
Yes	2.616	0.604	0.328–1.113	0.106
Annual visits to pediatrician				
1–2 times	5.307	0.604	0.393–0.928	0.021
3–4 times	4.446	0.582	0.352–0.963	0.035
PTHL-SR results				
Knowledge	12.243	0.718	0.597–0.865	<0.001
Understanding	2.215	0.858	0.702–1.050	0.137
Numeracy	2.973	0.850	0.705–1.026	0.090
Access to information	0.692	0.733	0.353–1.523	0.406
Total PTHL-SR result	0.868	0.868	0.795–0.947	0.002

## References

[1] Kaushal R., Bates D.W., Landrigan C., McKenna K.J., Clapp M.D., Federico F., Goldmann D.A. (2001). Medication errors and adverse drug events in pediatric inpatients. JAMA.

[2] Koster E.S., Philbert M., Bouvy L. (2015). Health literacy among pharmacy visitors in the Netherlands. Pharmacoepidemiol. Drug Saf..

[3] Ghanbari S., Rameyankhani A., Montazeri A., Mehrabi Z. (2016). Health Literacy Measure for Adolescents (HELMA): Development and Psychometric properties. PLoS ONE.

[4] Boztepe H., Özdemir H., Karababa Ç., Yıldız Ö. (2016). Administration of oral medication by parents at home. J. Clin. Nurs..

[5] Amiri S., Shafiee-Kandjani A.R., Noorazar S.G., Ivrigh S.R., Abdi S. (2016). Knowledge and attitude of parents of children with attention deficit hyperactivity disorder towards the illness. Iran. J. Psychiatry Behav. Sci..

[6] Gomez Dinger P.L., Kaplan M.S. (2008). The Impact of Parents’ Medication Beliefs on Asthma Management. Pediatrics.

[7] Bush P.J., Iannotti R.J. (1990). A children’s health belief model. Med Care.

[8] Chan V., Tran H. (2016). Purchasing OTC medicines from Australian pharmacy: What do the pharmacy customers value and expect?. Pharm. Pract..

[9] de Bont E.G., Francis N.A., Dinant G.J., Cals J.W. (2014). Parents’ knowledge, attitudes, and practice in childhood fever: An internet-based survey. Br. J. Gen. Pract..

[10] Ubavić S., Bogavac-Stanojević N., Jović-Vraneš A., Krajnović D. (2018). Understanding of information about medicines use among parents of pre-school children in Serbia: Parental pharmacotherapy literacy questionnaire (PTHL-SR). Int. J. Env. Res. Public Health.

[11] Hämeen-Anttila K., Halonen P., Siponen S., Holappa M., Ahonen R. (2011). Parental attitudes toward medicine use in children in Finland. Int. J. Clin. Pharm..

[12] Bailey S.C., Pandit A.U., Yin S., Federman A., Davis T.C., Parker R.M., Wolf M.S. (2009). Predictors of misunderstanding pediatric liquid medication instructions. Fam. Med..

[13] Yin H.S., Parker R.M., Sanders L.M., Mendelsohn A., Dreyer B.P., Bailey S.C., Patel D.A., Jimenez J.J., Kim K.Y., Jacobson K. (2017). Pictograms, Units and Dosing Tools, and Parent Medication Errors: A Randomized Study. Pediatrics.

[14] Torres A., Parker R.M., Sanders L.M., Wolf M.S., Bailey S.C., Patel D.A., Jimenez J.J., Kim K.Y., Dreyer B.P., Mendelsohn A.L. (2017). Parent Preferences and Perceptions of Milliliters and Teaspoons: Role of Health Literacy and Experience. Acad. Pediatrics.

[15] Milani G.P., Benini F., Dell’Era L., Silvagni D., Podestà A.F., Mancusi R.L., Fossali E.F. (2017). Acute pain management: Acetaminophen and ibuprofen are often under-dosed. Eur. J. Pediatrics.

[16] Ubavić S., Krajnovic D., Bogavac-Stanojevic N. (2018). Pharmacotherapy literacy (PTHL- SR) questionnaire for parents of pre-school children in Serbia: Construction and psychometric characteristics. Vojnosanit. Pregl..

[17] Tausch A.P., Menold N. (2016). Methodological Aspects of Focus Groups in Health Research. Glob. Qual. Nurs. Res..

[18] Sharts-Hopko N.C. (2001). Focus group methodology: When and why?. J. Assoc. Nurses Aids Care.

[19] Samojlik I., Mijatović V., Gavarić N., Krstin S., Božin B. (2013). Consumers’ attitude towards the use and safety of herbal medicines and herbal dietary supplements in Serbia. Int. J. Clin. Pharm..

[20] Gazibara T., Nikolovski J., Lakić A., Pekmezović T., Kisić-Tepavčević D. (2014). Parental knowledge, attitudes, and behaviors towards children with epilepsy in Belgrade (Serbia). Epilepsy Behav..

[21] Birchley N., Conroy S. (2002). Parental management of OTC medicines. Paediatr. Nurs..

[22] Vervloeta M., Van Dijk L., Rademakers J., Bouvy M.L., De Smet P.A.G.M., Philbert D., Koster E.S. (2018). Recognizing and Addressing Limited Pharmaceutical literacy: Development of the RALPH interview guide. Res. Soc. Adm. Pharm..

[23] Koster E.S., Philbert D., Van Dijk L., Rademakers J., De Smet P.A.G.M., Bouvy M.L. (2018). Recognizing pharmaceutical illiteracy in community pharmacy: Agreement between a practice-based interview guide and questionnaire based assessment. Res. Soc. Adm. Pharm..

[24] Stein L., Bergdahl M., Pettersen K.S., Bergdahl J. (2018). Effects of the Conceptual Model of Health Literacy as a Risk: A Randomised Controlled Trial in a Clinical Dental Context. Int. J. Environ. Res. Public Health.

